# Longitudinal Innate and Heterologous Adaptive Immune Responses to SARS‐CoV‐2 JN.1 in Transplant Recipients With Prior Omicron Infection: Limited Neutralization but Robust CD4^+^ T‐Cell Activity

**DOI:** 10.1111/tid.70067

**Published:** 2025-07-02

**Authors:** Victor H. Ferreira, Brandon Keith, Faranak Mavandadnejad, Alejandro Ferro, Sara Marocco, Golnaz Amidpour, Alexandra Kurtesi, Freda Qi, Anne‐Claude Gingras, Victoria G. Hall, Deepali Kumar, Atul Humar

**Affiliations:** ^1^ University Health Network (UHN) Ajmera Transplant Centre Toronto General Hospital Research Institute (TGHRI) Toronto Ontario Canada; ^2^ Department of Laboratory Medicine and Pathobiology University of Toronto Toronto Ontario Canada; ^3^ Lunenfeld‐Tanenbaum Research Institute Sinai Health Toronto Ontario Canada; ^4^ Department of Molecular Genetics University of Toronto Toronto Ontario Canada

**Keywords:** COVID‐19, humoral immunity, innate immunity, JN.1, Neutralizing antibodies, Omicron JN.1 subvariant, organ transplantation, SARS‐CoV‐2, T‐cells, transplant recipients

## Abstract

**Background:**

Solid organ transplant (SOT) recipients are at increased risk for severe COVID‐19 and often exhibit reduced vaccine efficacy due to chronic immunosuppression. As new SARS‐CoV‐2 variants emerge, understanding immune responses following natural infection remains critical for informing protection strategies in this vulnerable population. We conducted a longitudinal study of SOT recipients who had recovered from Omicron BA.1 or BA.2 infection, evaluating immune responses to the JN.1 subvariant at 4–6 weeks and 1 year postinfection.

**Methods:**

Neutralizing antibodies to JN.1 were measured using a pseudovirus neutralization assay, and JN.1‐specific T‐cell responses were assessed by flow cytometry. Frequencies of bulk T‐cells and innate immune cells, identified via flow cytometry, and their correlation with adaptive responses were also analyzed.

**Results:**

At 4–6 weeks, 30% of participants had detectable JN.1‐neutralizing antibodies, rising to 43% at one year, although titers remained low. In contrast, CD4⁺ T‐cell responses were robust and detected in 75%–83% of participants at 4–6 weeks, increasing to 75%–93% by 1 year. CD8⁺ T‐cell responses were observed less frequently. Exploratory correlations between innate and bulk T‐cell subsets with heterologous adaptive immune responses were investigated but did not reveal statistically significant relationships.

**Conclusion:**

These findings offer important insights into the durability and breadth of immunity following natural infection in immunocompromised transplant recipients. While heterologous neutralizing antibodies were limited, sustained CD4^+^ T‐cell responses may help mitigate severe disease following exposure to JN.1‐derived variants, which continue to dominate the SARS‐CoV‐2 landscape.

## Graphical abstract

In transplant recipients with prior Omicron infection, neutralizing antibodies against JN.1 were limited and often absent 1 year later. However, robust and durable CD4⁺ T‐cell responses were consistently observed, suggesting that cellular immunity may offer sustained protection against emerging SARS‐CoV‐2 variants, despite modest antibody responses.

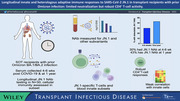

**Abbreviations**
BH: Benjamini–Hochberg
COVID‐19: coronavirus disease 2019
ID50: inhibitory dilution with 50% virus neutralization
IFN‐γ: interferon gamma
IL‐2: interleukin 2
IQR: interquartile range
NK: natural killer
PBMC: peripheral blood mononuclear cells
SARS‐CoV‐2: severe acute respiratory syndrome Coronavirus 2
SOT: solid organ transplant

## 1 Introduction

The initial waves of the Coronavirus disease 2019 (COVID‐19) pandemic saw the predominance of ancestral strains of severe acute respiratory syndrome Coronavirus 2 (SARS‐CoV‐2) along with its early variants like Alpha and Delta. With time, the virus evolved, leading to the emergence of subvariants like Omicron BA.1, with significant genetic differences, namely in the immunodominant spike protein, a major determinant of viral attachment and entry into host cells. Omicron subvariants have posed substantial challenges due to their enhanced transmissibility and ability to evade immune responses elicited by previous infection and immunization [1, 2]. Solid organ transplant (SOT) recipients are at elevated risk for severe outcomes of COVID‐19 [3], as well as experiencing vaccine failure [4, 5, 6, 7], mainly owing to the immunosuppression used to prevent organ rejection. Although the severity of COVID‐19 has decreased across variants over time in the SOT population, the rate of severe COVID‐19 during Omicron waves ranged 5.7%–16.1% [3], underscoring the continued threat that SARS‐COV‐2 poses in this uniquely vulnerable group.

Previous studies have highlighted the importance of neutralizing antibodies in protecting against SARS‐CoV‐2 infection and disease [8, 9, 10]. However, the durability and breadth of neutralizing antibody responses, including heterologous/cross‐neutralizing antibodies directed to newer subvariants, remain poorly understood in SOT recipients. T‐cell responses, which may be critical for mitigating severe disease from SARS‐CoV‐2 infection [11, 12, 13, 14], have been measured in SOT recipients, but mainly against ancestral SARS‐CoV‐2 antigens and mostly in the context of vaccination. Antibody and T‐cell longitudinal measurements are needed after SARS‐CoV‐2 infection to determine quality and durability of protection and the impact of factors including vaccination on shaping these immune responses. Although innate immune cells and bulk T‐cells may not necessarily provide information on antigen‐specific responses, their presence may offer critical information on an individual's broader immune‐competence. In addition, these cell types—which are generally easier to measure using conventional laboratory techniques—may serve as useful biological correlates of adaptive responses.

This study addresses critical knowledge gaps by investigating heterologous humoral and cellular immune responses in transplant recipients recovered from SARS‐CoV‐2 infection with Omicron BA.1 or BA.2 subvariants. We evaluated neutralizing antibodies and T‐cell responses to the JN.1 subvariant at two key time points: 4–6 weeks, and 1 year postinfection. JN.1 shares key spike mutations with early Omicron subvariants BA.1 and BA.2 but also harbors unique changes that enhance immune escape and ACE2 binding affinity [15]. Compared to ancestral strains, JN.1 is substantially more divergent, with over 30 amino acid changes in the spike protein alone, reflecting extensive antigenic evolution. While BA.1, BA.2, and JN.1 all fall under the broader Omicron umbrella, the genetic and antigenic distance between JN.1 and BA.1/BA.2 are significantly smaller than those between JN.1 and ancestral strains. Although JN.1 has now been superseded by newer subvariants, current circulating strains like LP.8.1 and XEC are derived from the JN.1 lineage [16, 17] and remain closely related. Thus, examining JN.1‐specific responses remains clinically relevant. Moreover, the longitudinal design of this study, evaluating both humoral and cellular immunity up to 1 year postinfection, offers real‐world insight into the durability and evolution of hybrid immunity in SOT recipients.

## 2 Methods

### 2.1 Study Cohort

Participants were enrolled from December 31, 2021 to February 01, 2022, coinciding with the emergence and dominance of Omicron BA.1 and BA.2 as circulating strains. All participants provided written informed consent to participate in the study and institutional research ethics board approval was obtained (University Health Network, CAPCR‐ID: 20‐5361). SARS‐CoV‐2 infection was confirmed via positive nasopharyngeal PCR; duration of PCR positivity was not determined. No participants had a prior diagnosis of COVID‐19 before participating in the study. Blood work was collected at 4–6 weeks and 1 year post COVID‐19 diagnosis for serum isolation. In a subset of participants, peripheral blood mononuclear cells (PBMCs) were collected and cryopreserved for bulk testing.

### 2.2 JN.1 Pseudovirus Neutralizing Antibodies Assay

Neutralizing antibodies against the spike protein of Omicron JN.1 (and earlier sub/variants [4]), were detected using a highly‐versatile pseudovirus neutralization assay, as described previously [18]. Briefly, lentiviral particles were prepared using a viral packaging vector, spike Omicron‐JN.1 constructs (generated from consensus sequences available from Outbreak.Info [19]), and the ZsGreen and luciferase reporter (pHAGE‐CMV‐Luc2‐IRES‐ZsGreen‐W, kindly provided by Jesse Bloom) in HEK293TN cells. Patient sera were serially diluted, followed by incubation with diluted virus at a 1:1 ratio for 1 h at 37°C prior to addition to cells. The final serum dilution ranged from 1:40 (lower limit of quantitation) to 1:24 414. The infected cells were lysed after 48 h, and luminescence (expressed in relative luciferase units) was measured using the BrightGlo Luciferase Assay System (Promega) and an EnVision 2015 Multimode Plate Reader (PerkinElmer). A sigmoidal curve was fitted to the data, and inhibitory dilution with 50% virus neutralization (ID50) titers were calculated using the nlsLM function from the minpack.lm package in R [20]. A value of one was assigned to undetectable neutralization responses, defined as those with no neutralization at the lowest dilution tested. The pseudovirus assay is a validated surrogate for live virus neutralization and has been shown to correlate with live‐virus PRNT assays for SARS‐CoV‐2 [18].

### 2.3 Measuring Cell‐Mediated Immunity Using Flow Cytometry

SARS‐CoV‐2 antigen‐specific T‐cell‐responses were assessed using peptide‐stimulated PBMCs and intracellular cytokine staining flow cytometry, as previously described [4, 21, 22]. Overlapping JN.1 spike peptides were incubated overnight with 10^6^ PBMCs in the presence of CD28/CD49d costimulatory antibodies (BD Biosciences) and a protein transport inhibitor (ThermoFisher Scientific) to prevent cytokine release. Overlapping peptides corresponded to the complete SARS‐CoV‐2 spike protein of Omicron JN.1 (JPT Peptide Technologies). Cells expressing interferon gamma (IFN‐γ) and interleukin 2 (IL‐2) either individually or simultaneously (polyfunctional) were measured. A minimum of 50 000 live CD3^+^ T‐cells were required for samples to be included in the flow analysis. Negative controls consisted of PBMCs incubated overnight with an equivalent percentage (0.2%) of dimethyl sulfoxide‐containing media. Cell Stimulation Cocktail (ThermoFisher) was used as a positive control, according to instructions. No fixed cutoff was applied to define positive responses. Instead, antigen‐specific T‐cell responses were defined by the presence of cytokine‐positive CD4⁺ or CD8⁺ T‐cells after background cytokine subtraction. This approach was chosen to preserve sensitivity in detecting low‐frequency responses, which are common in immunosuppressed populations and may still be biologically relevant. The following antibodies were used in the study: anti‐human CD3‐BV786, anti‐human CD4‐BUV805, anti‐human CD8‐BUV496, anti‐human IFN‐γ‐FITC, and anti‐human IL‐2‐APC. FVS 575 V was used as a viability marker. Additional antibodies included in our multiparameter flow cytometry panel to detect innate cell subsets were CD56‐BV711, CD68–BV421, CD16–BB700, CD14–PE, and CD11c–BUV661. All antibodies were purchased from BD Biosciences. Data was captured on a BD Symphony instrument in the UHN‐Sickkids Flow Cytometry Facility. Flow cytometry data were acquired using BD FacsDiva v6.1.3 and analyzed using FlowJo v10.7.1.

### 2.4 Statistical Analysis

Differences in the medians of paired observations were tested using the Wilcoxon matched‐pairs signed‐rank test, and unpaired data were compared using the Mann–Whitney *U* test. Differences in three or more paired observations were compared using Friedman's test with Dunne's correction for multiple comparisons. Differences in proportions were compared using the χ^2^ or Fisher's exact tests, as appropriate. Spearman correlation was used to find relationships between variables with the Benjamini–Hochberg (BH) method applied to correct for multiple comparisons. Adjusted *p* values < 0.05 were considered statistically significant. Statistical analyses and figure generation were performed using Prism version 10.4.1 (GraphPad Software).

## 3 Results

### 3.1 Study Cohort Description

We enrolled 75 SOT recipients who provided whole blood at 4–6 weeks post‐COVID‐19 with Omicron BA.1 or BA.2 to evaluate homotypic and heterologous antibody and T‐cell responses in a previous study [4]. From that cohort, 30 participants later provided 1 year postinfection follow‐up sera and constitute the main cohort investigated in this study (Figure S1). The median time from COVID‐19 diagnosis to the 4–6‐week bloodwork visit was 40 days (interquartile range [IQR]: 36–51) and the median time from COVID‐19 diagnosis to the 1 year follow‐up visit was 354 days (IQR: 345–368) (Table 1). PBMC were available from 12 participants at 4–6 weeks and from 16 participants at 1 year post‐COVID‐19.

**TABLE 1:** Study cohort demographics.

	*N* = 30 SOT recipients
Age at COVID‐19, median (IQR)	56.0 (44.8–62.5)
Male sex—*n* (%)	22 (73.3)
Type of transplant—*n* (%)	
Heart	1 (3.3)
Kidney	9 (30.0)
Kidney–liver	1 (3.3)
Kidney–pancreas	4 (13.3)
Liver	6 (20.0)
Lung	9 (30.0)
Time from transplant to COVID‐19, in years—median (IQR)	6.3 (2.5–9.9)
Time from most recent dose of SARS‐CoV‐2 vaccine to COVID‐19, in days—median (IQR)	91.5 (50.3–127.5)
No. of comorbidities—median (IQR)	2 (1–3)
Vaccine doses before COVID‐19–median (IQR)	3 (2.75–3)
Received sotrovimab—*n* (%)	15 (50.0)
Received remdesivir—*n* (%)	2 (6.7)
Received dexamethasone—*n* (%)	1 (3.3)
Severity of Illness—*n* (%)	
Mild	28 (93.3)
Moderate	2 (6.7)
Immunosuppressive Medications at COVID‐19—*n* (%)	
Tacrolimus	24 (80.0)
Cyclosporine	6 (20.0)
Mycophenolate	27 (90.0)
Prednisone	25 (83.3)
Reduction of immunosuppression after COVID‐19—*n* (%)	22 (73.3)
Received an additional dose of SARS‐CoV‐2 vaccine after COVID‐19—*n* (%)	10 (33.3)
Experienced re‐infection—*n* (%)	2 (6.7)

Abbreviations: COVID‐19, coronavirus disease 2019; IQR, interquartile range; SARS‐CoV‐2, severe acute respiratory syndrome Coronavirus 2.

The median age at the time of COVID‐19 for the 30 SOT recipients in our study was 56 years (IQR: 45–63) and participants were predominantly male (73.3%). Types of transplants received included one heart (3.3%), nine kidney (30.0%), one combined kidney–liver (3.3%), four kidney–pancreas (13.3%), six liver (20.0%), and nine lung (30.0%) transplants. The median time from transplantation to COVID‐19 diagnosis was 6.3 years (IQR: 2.5–9.9). The median number of vaccine doses (monovalent ancestral formulation) received before COVID‐19 was 3.0 (IQR: 2.8–3.0). Severity of COVID‐19 illness was mostly mild (93.3%) with two participants experiencing moderate COVID‐19 comprising treatment with supplemental oxygen, pneumonia, and hospitalization for 4 or 5 days. Antiviral interventions in this cohort included sotrovimab, received by half (*n* = 15), remdesivir (6.7%), and dexamethasone (3.3%). At the time of COVID‐19, immunosuppressive agents used included tacrolimus (80.0%), cyclosporine A (20%), mycophenolate (90.0%), and prednisone (83.3%). A total of 22 (73.3%) participants had a reduction in their immunosuppression as an intervention following COVID‐19, with mycophenolate being the most commonly reduced agent.

The median time from most recent vaccination to breakthrough Omicron BA.1/BA.2 infection was 91.5 days (IQR: 50.3–127.5). Ten SOT recipients (33.3%) received an additional dose of SARS‐CoV‐2 vaccine in the year following COVID‐19 infection; five received the original monovalent formulation, three received the BA.1 bivalent vaccine, one received the BNT162b2 XBB.1.5 monovalent update, and one could not recall. Two participants experienced reinfection; both episodes were mild and occurred 230 and 312 days after their initial episodes of COVID‐19. Typing data for these two patients was not performed, but at the time of reinfection, the dominant subvariants in Toronto, Ontario, were BA.4/BA.5 (August 2022) and BQ.1.1 (November 2022).

### 3.2 Heterologous JN.1 Neutralizing Antibodies

**FIGURE 1:**
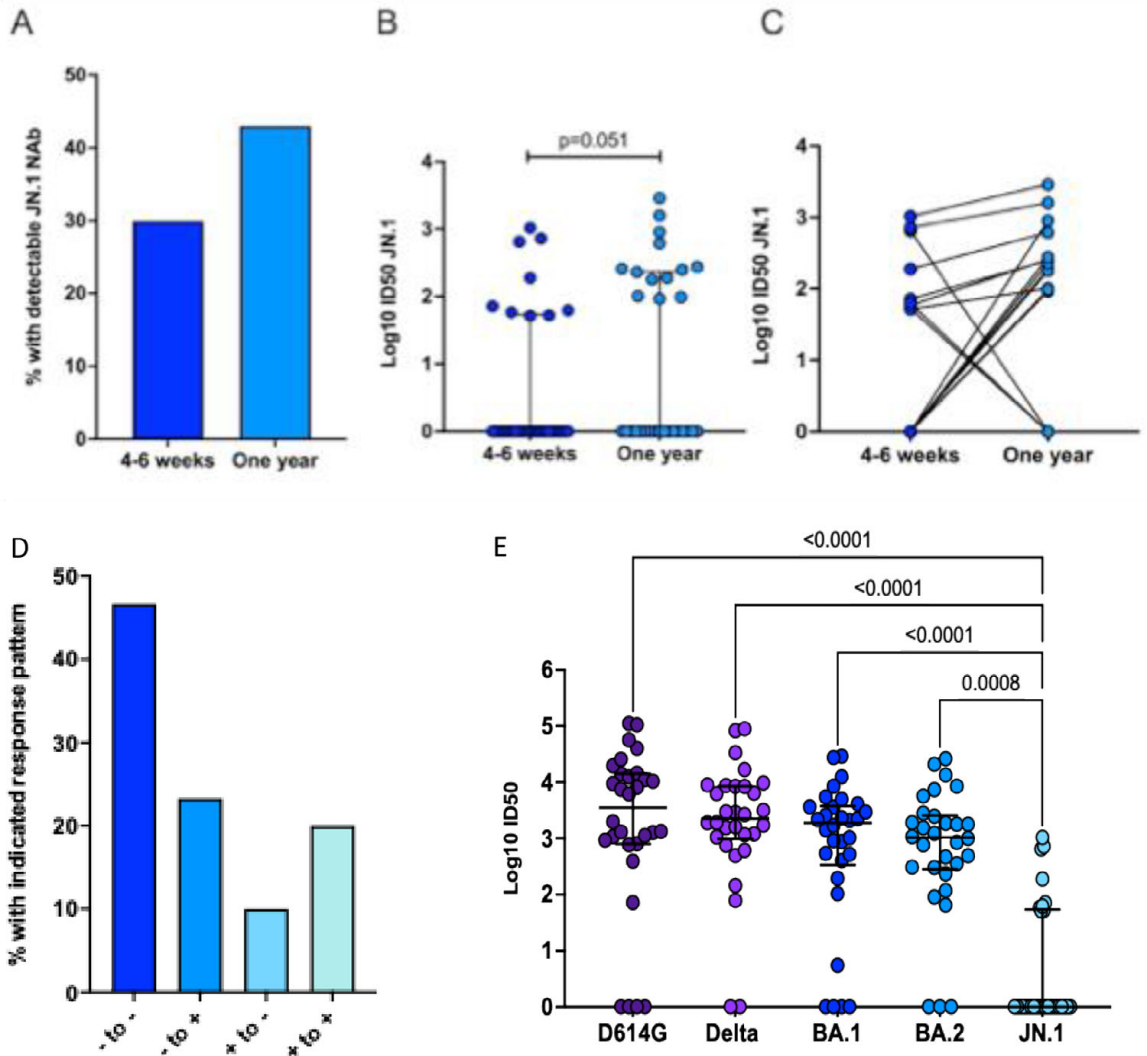
Neutralizing antibodies levels at 4–6 weeks and 1 year post‐COVID‐19. Thirty patients were compared at 4–6 weeks and 1 year postinfection with Omicron BA.1 or BA.2 and evaluated for cross‐neutralizing JN.1 neutralizing antibodies. (A) Proportion with detectable responses at both timepoints. (B) Box and whisker plot showing differences in neutralizing antibody levels between the visits. Median neutralizing antibodies levels were compared using the Wilcoxon matched‐pairs signed rank test. (C) Before and after dot plot showing changes over time for all participants in the study. Paired specimens are connected by lines. (D) Breakdown of neutralizing antibody detection across visits, showing the proportion of participants who stayed negative (− to −), went from negative to positive (− to +) or positive to negative (+ to −), or stayed positive (+ to +). (E) Thirty patients were tested for neutralizing antibodies responses against ancestral strain (D614G), Delta variant, and Omicron BA.1, BA.2, and JN.1 subvariants. Median titers were compared using Friedman's paired test with Dunne's correction for multiple comparisons. Adjusted *p* values relative to the JN.1 response are indicated above each bracket. ID50: inhibitory dilution with 50% virus neutralization, NAb: neutralizing antibody.

We define “homotypic” immune responses as those directed against the same SARS‐CoV‐2 variant responsible for the index infection (E.g., Omicron BA.1/BA.2 in this study), whereas “heterologous” responses refer to immune responses directed against antigenically distinct variants, in this case, the JN.1 subvariant, to which participants had not been previously exposed [4]. At 4–6 weeks post‐SARS‐CoV‐2 infection with Omicron subvariant BA.1/BA.2, nine (30.0%) individuals had detectable heterologous neutralizing antibodies to JN.1 (Figure 1A). The median log_10_ ID50 neutralizing level increased from 4–6 weeks to 1 year postinfection (Figure 1B,C), but this change was not statistically significant (*p* = 0.051). At the 1 year timepoint, the proportion of individuals with JN.1 neutralizing antibodies increased to 43.3% (Figure 1A). Seven (23.3%) participants gained heterologous antibodies over time, and three (10%) lost cross‐neutralization (Figure 1D). Among the six (20%) with detectable JN.1 neutralization at both timepoints, all had higher heterologous neutralizing antibody levels at the 1 year timepoint. A total of 14 (46.7%) SOT recipients remained undetectable for JN.1 neutralizing antibodies across study visits.

Univariate analysis of clinical and demographic variables was performed to identify factors associated with detectable heterologous JN.1 neutralization at 4–6 weeks (Table S1) and 1 year (Table S2) postinfection. However, no variables were significantly different between groups, including additional vaccine doses received after COVID‐19, reinfection, monoclonal antibody treatment, and changes in immunosuppression. Among the five participants with detectable JN.1 responses at 1 year that also received an additional dose of vaccine after having COVID‐19, three received an additional dose of the original monovalent formulation after experiencing COVID‐19; one received the BNT162b2 Pfizer XBB.1.5 monovalent update and one could not recall.

JN.1 median neutralizing antibody titers were compared to prior neutralizing antibody data generated from each patient against Omicron BA.1 and BA.2—representing the homotypic strains—along with D614G (ancestral strain), synonymous with early vaccine formulations, and the virulent Delta strain (Figure 1E). Antibodies were compared using the 4–6 weeks sample as 1 year neutralization testing for BA.1, BA.2, D614G, and Delta was not previously performed. Median levels of heterologous JN.1 antibodies were significantly lower than all other variants or sub‐variants tested. JN.1 heterologous responses correlated well with all neutralizing antibody responses measured, with the strongest correlation in antibody levels occurring with ancestral strain of SARS‐CoV‐2 (Figure S2; Spearman *r* = 0.76, BH adjusted *p* < 0.001). JN.1 heterologous responses also significantly correlated with neutralizing antibody levels against BA.1 and BA.2 subvariants (Vs. BA.1: Spearman *r* = 0.53, BH adjusted *p* = 0.0025; Vs. BA.2: Spearman *r* = 0.63, BH adjusted *p* < 0.001).

### 3.3 Heterologous JN.1‐Specific T‐Cell Responses

**FIGURE 2:**
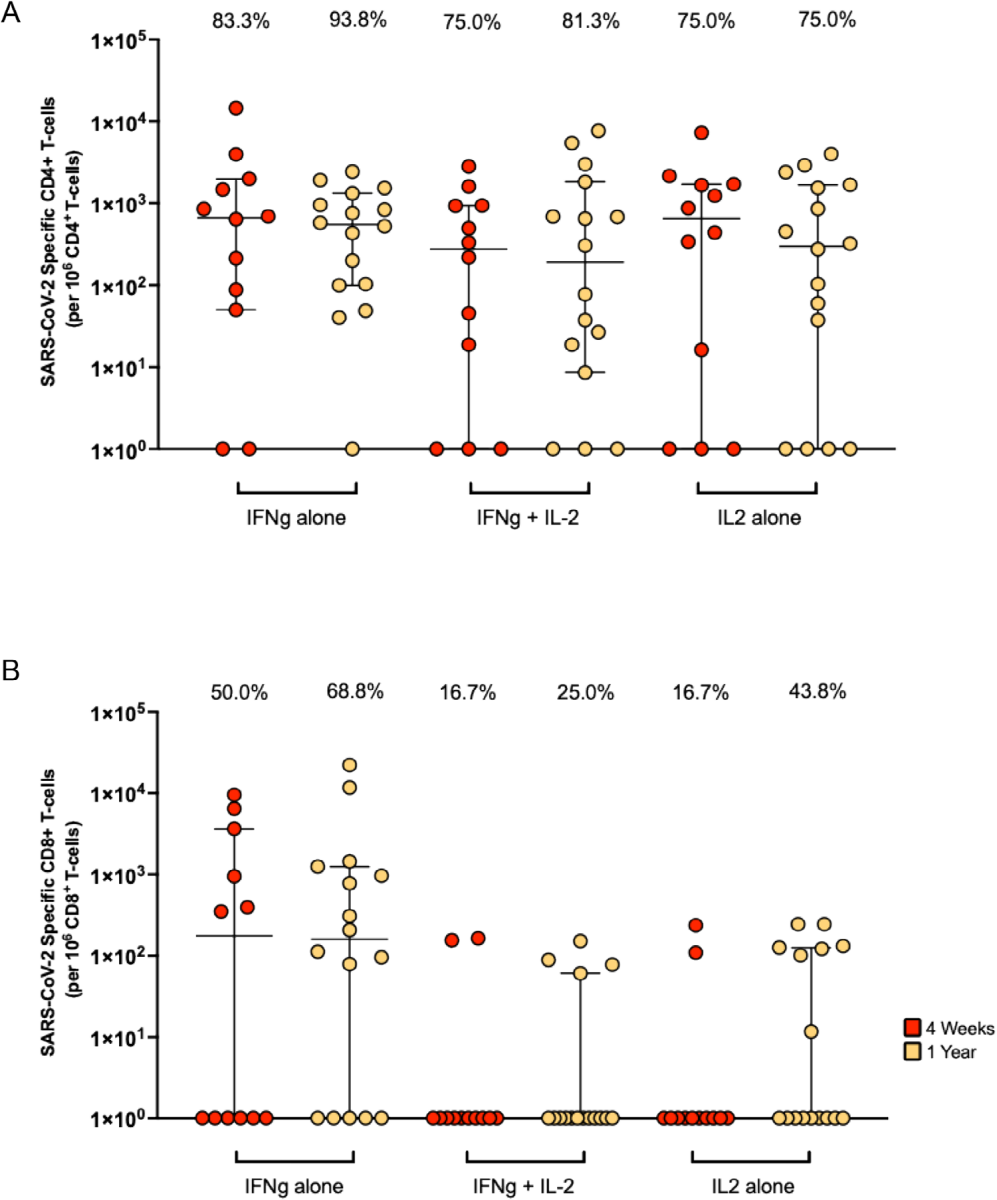
JN.1 specific T‐cell responses at 4–6 weeks and 1 year post‐COVID‐19. (A) CD4^+^ and (B) CD8^+^ T‐cell responses shown according to functional status of cells (IFN‐γ/IL‐2 mono‐ and polyfunctional cells) with the label along the bottom indicating the subset. The percentage reported above each group indicates the proportion of individuals with detectable T‐cell responses for that specific subset. Horizontal lines indicate the median for each group and vertical lines indicate the 95% confidence intervals. IFNg: interferon gamma, IL2: interleukin 2, SARS‐CoV‐2: severe acute respiratory syndrome Coronavirus 2.

We next examined differences in the T‐cell compartment, focusing on heterologous JN.1‐specific T‐cell responses measured using intracellular cytokine staining flow cytometry. A representative gating strategy is provided in Figure S3. In comparison with neutralizing antibody responses, T‐cell responses were readily detected. At 4–6 weeks post BA.1/BA.2 infection, 75.0%–83.3% of participants (depending on the T‐cell functional subset) had detectable heterologous CD4^+^ T‐cell responses directed to this contemporary subvariant (Figure 2A); 9/12 (75.0%) of participants had detectable JN.1‐responsive polyfunctional CD4^+^ T‐cells, and 10/12 (83.3%) had JN.1‐responsive IL‐2 monofunctional CD4^+^ T‐cells. At the 1 year timepoint, the proportion of individuals with JN.1‐specific CD4^+^ T‐cell responses ranged 75.0%–93.8%. At both timepoints, the most commonly identified antigen‐specific CD4^+^ T‐cell subset was IFN‐γ monofunctional cells. JN.1‐specific CD4^+^ and CD8^+^ T‐cell subset frequencies were higher at 1 year, but this was not statistically significant (*p* > 0.05). CD8^+^ T‐cell responses were less commonly detected (Figure 2B). JN.1 specific CD8^+^ T‐cell subsets were found in 16.7%–50.0% of participants at 4–6 weeks and 25.0%–68.8% at 1‐year, and similar to the CD4^+^ response, the main subset of JN.1‐responsive CD8^+^ T‐cells detected were IFN‐γ monofunctional T‐cells which were found in 6/12 and 11/16 SOT recipients at 4–6 weeks and 1 year, respectively.

### 3.4 Correlations Between Bulk T‐Cells and Innate Cells in Blood With Heterologous JN.1 Specific T‐Cell and Neutralizing Antibody Responses

**FIGURE 3:**
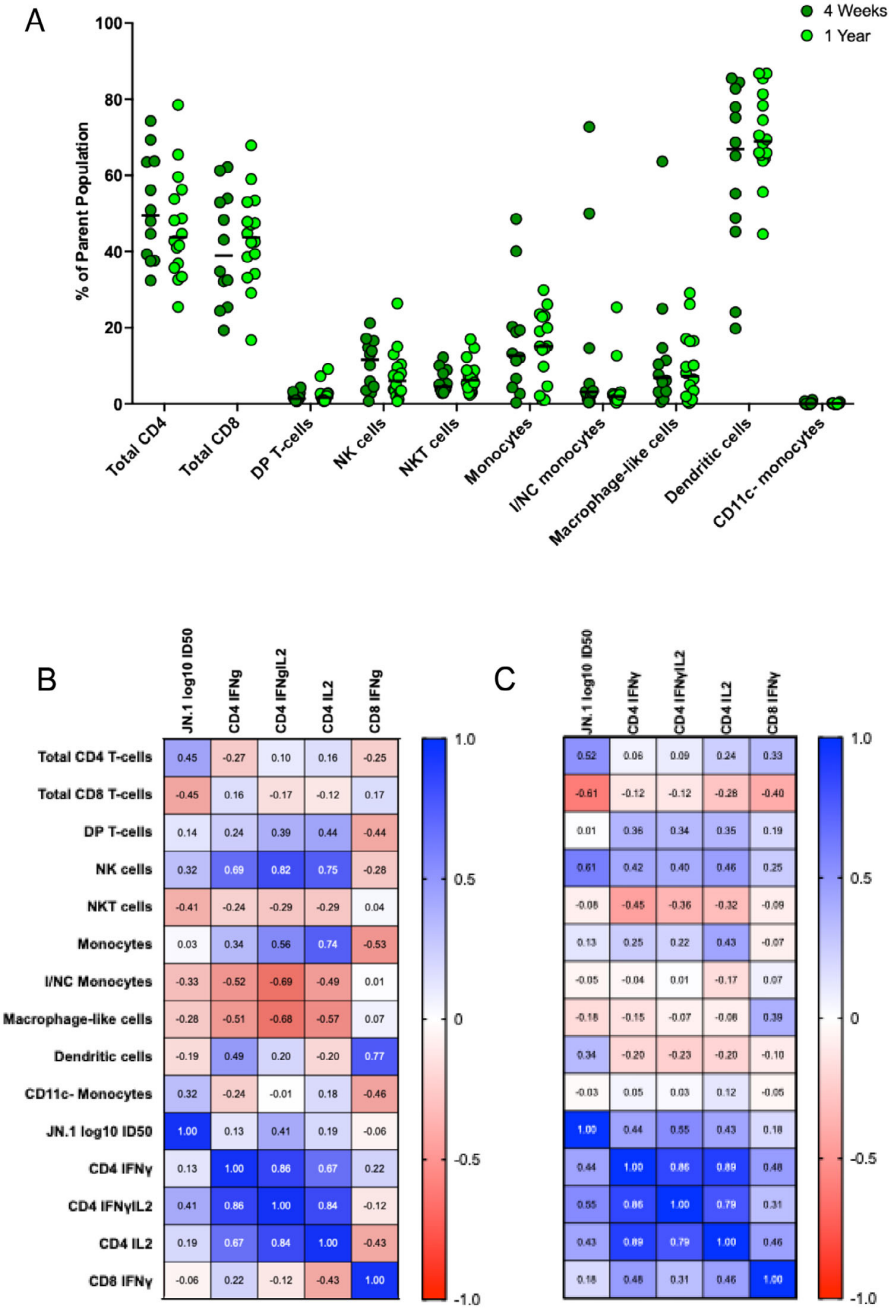
Bulk T‐cell and innate cell frequencies in peripheral blood and correlation with antigen‐specific T‐cell and neutralizing antibody responses. (A) Frequencies (relative to parental populations) are shown with the horizontal line indicating the median frequency. Correlation matrix of innate cell and bulk T‐cell responses at 4–6 weeks (B) and 1 year (C) with heterologous JN.1‐specific adaptive responses. DP: double positive, I/NC: intermediate/nonclassical, ID50: inhibitory dilution with 50% virus neutralization, NK: natural killer.

In patients with available PBMC for testing, we additionally enumerated frequencies of three bulk T‐cell populations (CD4^+^, CD8^+^, and CD4^+^CD8^+^/double positive T‐cells) and seven innate cell subsets in peripheral blood: natural killer (NK) cells, NKT cells, monocytes, CD11c^‐^ monocytes, intermediate/nonclassical monocytes, macrophage‐like cells [23], and dendritic cells (Figure S3). Frequencies of these 10 subsets are shown in Figure 3A and were overall stable between visits. JN.1‐specific CD4^+^ T‐cell subsets showed modest correlation with each other at 4–6 weeks, but strong correlation with each other at 1 year postinfection (Figure 3B,C; 1 year postinfection—IFN‐γIL‐2 vs. IFN‐γ, Spearman *r* = 0.86, BH adjusted *p* = 0.0020; IFN‐γIL‐2 vs. IL‐2, Spearman *r* = 0.79, BH adjusted *p* = 0.014; IFN‐γ vs. IL‐2, Spearman *r* = 0.89, BH adjusted *p* < 0.001). Overall, innate and bulk T‐cell subsets did not correlate with heterologous JN.1 antibody or T‐cell responses at either time point.

## 4 Discussion

This is the first study to evaluate JN.1 cross‐reactive humoral and cellular immune responses in SOT recipients. We also provide longitudinal data spanning 1 year and assess how evolving factors during the pandemic—such as vaccine type, additional doses, and reinfection—and contemporaneous innate cell and bulk T‐cell subsets, influenced heterologous immunity. Overall, JN.1‐specific neutralization rates were low compared to earlier variants at both 4–6 weeks and 1 year postinfection. Interestingly, a modest increase in neutralization was observed at 1 year, which could not be explained by expected factors such as vaccination or reinfection. In contrast, heterologous CD4^+^ T‐cell responses, including polyfunctional CD4^+^ T‐cells, were robust and frequently detected at both timepoints, suggesting a role in protection against future JN.1‐related disease. Although JN.1 has since been displaced by newer sublineages, their close genetic relationship to JN.1 underscores the continued clinical relevance of our findings.

A few studies in non‐transplant populations have investigated heterologous neutralizing and/or T‐cell responses at 1‐year postinfection, however, were mainly performed in those recovered from infection with pre‐Omicron strains. In these studies, neutralizing antibody levels generally declined over a 1‐year timepoint, including cross‐neutralizing antibodies to Omicron subvariants, however T‐cells remained highly cross‐reactive [24, 25]. Our T‐cell data in this and prior studies is largely in concordance with this [4, 26], but our antibody data contrasted showing some increase in cross‐neutralization over time, with nearly half of SOT recipients having detectable heterologous antibodies at 1‐year. This finding remains unexplained by our analysis, but may be due to multiple factors, working in conjunction and on different levels, including reduction in immunosuppression [27, 28] in the year following infection, additional doses and formulations of vaccines, reinfection—whether apparent or inapparent—and use of antiviral treatments.

While neutralizing antibodies are important for preventing infection and symptomatic disease, T‐cells are believed to play a critical role in limiting severe COVID‐19 [11], especially in the face of emerging variants. However, definitive correlates of T‐cell protection remain elusive [21, 29]. Although mutations in the Omicron BA.1 variant significantly impair serum neutralization compared to earlier strains, studies in immunocompetent individuals suggest that cellular immunity is more conserved: up to 70%–80% of ancestral CD4^+^ and CD8^+^ T‐cell responses are maintained in those vaccinated or previously infected with pre‐Omicron variants [30, 31]. In our current study of SOT recipients, most individuals exhibited detectable antigen‐specific T‐cell responses. Notably, IFN‐γ‐producing CD4^+^ T‐cells were the most abundant subset. When we previously examined homotypic T‐cell responses following COVID‐19 in SOT recipients (E.g., ancestral responses following ancestral infection or BA.1 responses following BA.1 infection), we observed a hierarchy: IL‐2 monofunctional > polyfunctional > IFN‐γ monofunctional CD4^+^ subsets [4, 21]. However, with respect to heterologous responses, including those in this study, we found overall lower frequencies of all T‐cell subsets, with the relative abundance pattern shifted. Specifically, IFN‐γ monofunctional cells became the dominant subset, and frequencies across all subsets were more uniform. This may reflect a fundamental difference in the quality and functionality of these responses, including reduced polyfunctionality of heterologous CD4^+^ T‐cells. While this remains a hypothesis, it underscores the need for future studies to explore the functional implications and clinical consequences of these altered response profiles, particularly in immunocompromised populations.

Our study had several limitations. The limited sample size and incomplete specimen availability reduced our statistical power to detect associations between clinical variables and immune responses, and likely contributed to nonsignificant findings in exploratory subgroup analyses, including additional vaccine doses received after COVID‐19, reinfection, monoclonal antibody treatment, and changes in immunosuppression. Further, the cohort was predominantly male (73%), which might skew results, particularly as sex‐based differences in COVID‐19 infection outcomes and vaccination have been documented [32, 33]. In addition, variability in timing and type of postinfection vaccination could have influenced measured immune responses, particularly heterologous neutralization, although our sample size precluded meaningful subgroup analysis. We did not apply a fixed threshold to define T‐cell positivity, as low‐frequency responses were common and potentially meaningful in this immunosuppressed cohort. However, this approach may limit comparability across studies that use standardized cutoffs and should be interpreted accordingly. It is also important to note that flow cytometry‐based T‐cell assays, while valuable for research, are not routinely available in clinical laboratories, limiting their immediate applicability for patient care. We also cannot rule out that JN.1 responses at 1 year may have been influenced by subclinical reinfections that we were unable to capture in our study. Lastly, while our study focuses on the JN.1 variant, which has since been supplanted by newer SARS‐CoV‐2 subvariants, it remains clinically relevant. Few studies have examined JN.1‐directed immune responses, particularly in immunocompromised individuals. Notably, current variants are direct descendants of JN.1 and share significant genetic similarities. This genetic continuity suggests that our findings on JN.1‐specific immune responses may offer insights into responses to these newer strains. However, caution is warranted when extrapolating these results, as mutations in the spike protein can affect neutralizing antibody efficacy, while T‐cell epitopes tend to be more conserved, potentially preserving T‐cell recognition across variants. Therefore, our data may be particularly informative regarding T‐cell–mediated immunity to current strains, though ongoing surveillance and research are essential to fully understand these dynamics.

Strengths of our study included an in‐depth analysis of cellular immunity, including longitudinal timepoints, and heterologous neutralizing antibody responses against contemporaneously relevant subvariants. Clinically, this knowledge underscores the importance of tailored vaccine strategies for vulnerable populations, especially as emerging variants continue to evolve. Identifying persistent CD4^+^ T‐cell responses and potential innate immune biomarkers may help clinicians identify patients with suboptimal adaptive responses. These findings could inform monitoring strategies, individualized vaccine schedules, and the development of immunomodulatory therapies that enhance both humoral and cellular immunity.

## Author Contributions

**Victor H. Ferreira**: conceptualization, investigation, funding acquisition, writing – original draft, methodology, visualization, writing – review and editing, formal analysis, project administration, supervision. **Brandon Keith**: investigation, writing – review and editing. **Faranak Mavandadnejad**: investigation, writing – review and editing. **Alejandro Ferro**: investigation, writing – review and editing. **Sara Marocco**: investigation, writing – review and editing. **Golnaz Amidpour**: investigation, writing – review and editing. **Alexandra Kurtesi**: investigation, writing – review and editing, formal analysis. **Freda Qi**: investigation, writing – review and editing, formal analysis. **Anne‐Claude Gingras**: methodology, writing – review and editing, supervision. **Victoria G. Hall**: methodology, formal analysis, writing – review and editing, investigation. **Deepali Kumar**: conceptualization, funding acquisition, methodology, writing – review and editing, formal analysis, project administration, supervision. **Atul Humar**: conceptualization, funding acquisition, methodology, writing – review and editing, formal analysis, project administration, supervision.

## Conflicts of Interest

The authors declare no conflicts of interest.

## Supporting information




**Supplementary Table 1: Factors associated with detectable JN.1 specific neutralizing responses at 4‐6 weeks post COVID‐19. Supplementary Table 2: Factors associated with detectable JN.1 specific neutralizing responses at one year post COVID‐19. Supplementary Figure 1: Study overview**. A total of 75 solid organ transplant (SOT) recipients were enrolled and provided serum at 4‐6 weeks post‐Omicron BA.1 or BA.2 infection. Details of this cohort are described elsewhere. From this group, 30 participants provided serum at one year post‐infection, comprising the main study group. Additionally, 12 and 16 participants also provided peripheral blood mononuclear cells (PBMC) at 4‐6 weeks and one year post‐infection, respectively.
**Supplementary Figure 2: Correlation matrix of spearman correlations between JN.1 and other sub‐variants**. The legend represents the value of the Spearman correlation coefficient for each comparison using the log10 ID50 levels for each variant, which is also shown for each respective comparison. Asterisks (*) indicate statistically significant correlations with JN.1 titres after correction for multiple comparisons using Benjamini‐Hochberg method.
