# Supplementary material for: Longitudinal Innate and Heterologous Adaptive Immune Responses to SARS‐CoV‐2 JN.1 in Transplant Recipients With Prior Omicron Infection: Limited Neutralization but Robust CD4+ T‐Cell Activity
Source: Transpl Infect Dis. 2025 Jul 2;27(4):e70067. doi: 10.1111/tid.70067 (PMC12416463; doi:10.1111/tid.70067)
Supplement: Supplementary file 1 — Supplementary Table 1: Factors associated with detectable JN.1 specific neutralizing responses at 4‐6 weeks post COVID‐19. Supplementary Table 2: Factors associated with detectable JN.1 specific neutralizing responses at one year post COVID‐19. Supplementary Figure 1: Study overview. A total of 75 solid organ transplant (SOT) recipients were enrolled and provided serum at 4‐6 weeks post‐Omicron BA.1 or BA.2 infection. Details of this cohort are described elsewhere. From this group, 30 participants provided serum at one year post‐infection, comprising the main study group. Additionally, 12 and 16 participants also provided peripheral blood mononuclear cells (PBMC) at 4‐6 weeks and one year post‐infection, respectively. Supplementary Figure 2: Correlation matrix of spearman correlations between JN.1 and other sub‐variants. The legend represents the value of the Spearman correlation coefficient for each comparison using the log10 ID50 levels for each variant, which is also shown for each respective comparison. Asterisks (*) indicate statistically significant correlations with JN.1 titres after correction for multiple comparisons using Benjamini‐Hochberg method. [file TID-27-e70067-s001.docx]

**Supplementary Table 1 - Factors associated with detectable JN.1 specific neutralizing responses at 4-6 weeks post COVID-19.**

|  | JN.1 neutralizing antibodies detected,  n=9 | JN.1 neutralizing antibodies not detected, n=21 | Univariate p-value |
| --- | --- | --- | --- |
| Age at COVID-19, in years – median (IQR) | 53 (47-64) | 57 (38-63) | 0.92 |
| Male Sex – n (%) | 9 (100) | 13 (61.9) | 0.067 |
| Type of transplant – n (%)  Heart  Kidney  Kidney-lung  Kidney-pancreas  Liver  Lung | 0 (0.0)  3 (33.3)  1 (11.1)  0 (0.0)  3 (33.3)  2 (22.2) | 1 (4.8)  6 (28.6)  0 (0.0)  4 (19.0)  3 (14.3)  7 (33.3) | 0.33 |
| 2 or more co-morbidities – n (%) | 8 (88.9) | 11 (52.4) | 0.10 |
| Sotrovimab treatment – n (%) | 3 (33.3) | 12 (57.1) | 0.43 |
| Time from transplant to COVID, in years – median (IQR) | 8.8 (4.3-13.0) | 5.4 (2.5-7.9) | 0.13 |
| Reduction of immunosuppression – n (%) | 5 (55.6) | 17 (81.0) | 0.20 |
| Immunosuppression at COVID-19 – n (%)  Tacrolimus  Cyclosporine  Mycophenolate  Prednisone | 7 (77.8)  2 (22.2)  8 (88.9)  7 (77.8) | 17 (81.0)  4 (19.0)  20 (95.2)  18 (85.7) | >0.99  >0.99  0.52  0.62 |
| Immunosuppressive levels/dose – median (IQR)  Tacrolimus level  Cyclosporine level  Mycophenolate dose mg  Prednisone dose mg | 8.1 (5.5-9.9)  305 (118-491)  1260 (450-1440)  5.0 (5.0-7.5) | 7.4 (6.6-11.0)  136 (111-555)  1080 (720-1440)  5.0 (5.0-7.5) | 0.59  >0.99  0.89  0.70 |
| Number of vaccine doses prior to COVID-19 – n (%)  0  1  2  3  4 | 0 (0.0)  0 (0.0)  1 (11.1)  8 (88.9)  0 (0.0) | 2 (9.5)  0 (0.0)  4 (19.0)  13 (61.9)  2 (9.5) | 0.53 |

Abbreviations: COVID-19 – coronavirus disease 2019, IQR – interquartile range, mg – milligrams.

**Supplementary Table 2 - Factors associated with detectable JN.1 specific neutralizing responses at one year post COVID-19.**

|  | JN.1 neutralizing antibodies detected,  n=13 | JN.1 neutralizing antibodies not detected, n=17 | Univariate p-value |
| --- | --- | --- | --- |
| Age at COVID-19, in years – median (IQR) | 56 (49-66) | 56 (38-61) | 0.39 |
| Male Sex – n (%) | 10 (76.9) | 12 (70.6) | >0.99 |
| Type of transplant – n (%)  Heart  Kidney  Kidney-lung  Kidney-pancreas  Liver  Lung | 0 (0.0)  6 (46.2)  0 (0.0)  2 (15.4)  2 (15.4)  3 (23.1) | 1 (5.9)  3 (17.6)  1 (5.9)  2 (11.8)  4 (23.5)  6 (35.3) | 0.64 |
| 2 or more co-morbidities – n (%) | 10 (76.9) | 9 (52.9) | 0.26 |
| Sotrovimab treatment – n (%) | 5 (38.6) | 10 (58.8) | 0.46 |
| Received tixagevimab/cilgavimab – n (%) | 2 (15.4) | 5 (29.4) | 0.67 |
| Time from transplant to COVID, in years – median (IQR) | 6.4 (2.3-11.0) | 5.5 (3.0-9.4) | 0.87 |
| Immunosuppression at 1-year – n (%)  Tacrolimus  Cyclosporine  Mycophenolate  Prednisone | 10 (76.9)  3 (23.1)  11 (84.6)  11 (84.6) | 14 (82.4)  3 (17.6)  13 (76.5)  13 (76.5) | >0.99  >0.99  0.67  0.67 |
| Had a change in their immunosuppression after COVID-19? – n (%) | 10 (76.9) | 12 (70.6) | >0.99 |
| Immunosuppressive levels/dose at 1-year – median (IQR)  Tacrolimus level  Cyclosporine level  Mycophenolate dose mg  Prednisone dose mg | 6.8 (6.2-7.5)  99 (94-330)  720 (360-1440)  5.0 (5.0-7.5) | 8.6 (5.8-13.0)  133 (121-148)  900 (630-1440)  5.0 (5.0-5.0) | 0.24  0.70  0.53  0.30 |
| Received additional vaccine dose after COVID – n (%) | 5 (38.5) | 5 (29.4) | 0.44 |
| Total number of vaccine doses at one-year post-infection – n (%)  0  1  2  3  4  5 | 0 (0.0)  0 (0.0)  0 (0.0)  8 (61.5)  5 (38.5)  0 (0.0) | 2 (11.8)  0 (0.0)  4 (23.5)  6 (35.3)  4 (23.5)  1 (5.9) | 0.14 |
| COVID-19 re-infection – n (%) | 2 (15.4) | 0 (0.0) | 0.18 |
| Time from most recent vaccination to one-year bloodwork, in days - median (IQR)^a^ | 406 (234-432) | 466 (180-512) | 0.22 |

Abbreviations: COVID-19 – coronavirus disease 2019, IQR – interquartile range, mg – milligrams.

**Supplementary Figure 1**

**Supplementary Figure 1 – Study overview.** A total of 75 solid organ transplant (SOT) recipients were enrolled and provided serum at 4-6 weeks post-Omicron BA.1 or BA.2 infection. Details of this cohort are described elsewhere. From this group, 30 participants provided serum at one year post-infection, comprising the main study group. Additionally, 12 and 16 participants also provided peripheral blood mononuclear cells (PBMC) at 4-6 weeks and one year post-infection, respectively.

**Supplementary Figure 2**

***

***

***

**

***

**Supplementary Figure 2 - Correlation matrix of spearman correlations between JN.1 and other sub-variants.** The legend represents the value of the Spearman correlation coefficient for each comparison using the log10 ID50 levels for each variant, which is also shown for each respective comparison. Asterisks (*) indicate statistically significant correlations with JN.1 titres after correction for multiple comparisons using Benjamini-Hochberg method. **=p<0.01, ***=p<0.001.

**Supplementary Figure 3**

**Supplementary Figure 3 – Gating strategy used to identify different cell subsets.** Top row shows monocyte-associated gating. Live, single cells were first identified and then characterized in terms of CD14 vs CD11c expression. The main CD14^+^ population (which was also CD11c^+^) were defined as the “monocytes” and further analyzed in terms of CD68 and CD16 expression. The bottom row shows the approach taken to identify lymphocytes. Live, single cells were gated in terms of CD3 vs CD56 expression. The CD3^+^CD56^-^ cells (T-cells) were further characterized in terms of CD4 vs CD8 expression. Antigen-specific T-cell responses based on intracellular cytokine staining of interferon-gamma (IFN-γ) and interleukin-2 (IL-2) expression in peptide-stimulated and unstimulated (control) peripheral blood mononuclear cells.
